# Crystal structure of fuco­xanthin: resolving a long-standing crystallographic challenge

**DOI:** 10.1107/S2053229626008946

**Published:** 2026-09-05

**Authors:** Yui Wakasa, Mao Minoura

**Affiliations:** a Department of Chemistry, College of Science, Rikkyo University, 3-34-1 Nishi-Ikebukuro, Toshima-ku, Tokyo 171-8501, Japan; J-PARC Center, Japan Atomic Energy Agency, Japan

**Keywords:** crystal structure, fuco­xanthin, carotenoid, allene, synchrotron radiation, natural product

## Abstract

Fucoxanthin (C_42_H_58_O_6_) is a highly functionalized allenic ep­oxy xanthophyll containing hy­droxy, acet­oxy, ketone and conjugated polyene units. Although its constitution and stereochemistry had been established by chemical, spectroscopic and synthetic studies, its small-mol­ecule single-crystal X-ray structure has remained unavailable. Moss reported in 1979 [*Pure Appl. Chem.* (1979), **51**, 507–514] that an early X-ray study by G. M. Sheldrick had reached only a partial solution of the fuco­xanthin structure and that refinement had stalled at *R* ≃ 25%. The first small-mol­ecule single-crystal X-ray structure of fuco­xanthin has now been determined as the diethyl ether monosolvate, C_42_H_58_O_6_·C_4_H_10_O, at 100 K in the space group *P*1 using synchrotron radiation (λ = 0.4132 Å). The asymmetric unit contains two crystallographically independent fuco­xanthin mol­ecules and two diethyl ether mol­ecules. The structure defines the relative stereochemistry, allene geometry, epoxide geometry, polyene bond-length alternation and solvate-assisted packing of fuco­xanthin, and provides an independent small-mol­ecule metrical reference for com­parison with protein-bound fuco­xanthin ligands.

## Abstract 2

The first small-mol­ecule single-crystal X-ray structure of fuco­xanthin is reported, resolving a long-standing crystallographic problem in carotenoid structural chemistry.

## Introduction

Fucoxanthin is an oxygenated C_42_ carotenoid of the xanthophyll family and one of the characteristic pigments of brown algae and diatoms (Mohibbullah *et al.*, 2022 ▸; Nogueira *et al.*, 2025 ▸). In the structural nomenclature of carotenoids, fuco­xanthin is a highly functionalized β,β-carotenoid derivative containing hy­droxy, acet­oxy, ep­oxy, ketone and allenic units. It was first isolated from brown algae in the early 20th century (Willstätter & Page, 1914 ▸), and its unusual combination of functional groups has made it a persistent subject in carotenoid structural chemistry.

The constitution of fuco­xanthin was established before the routine small-mol­ecule crystallographic analysis of extended carotenoids became practical. Fucoxanthin and related pig­ments were reported in early chemical and spectroscopic studies (Bonnett *et al.*, 1966 ▸), and the detailed structure and reactions of fuco­xanthin were subsequently formulated as 3′-acet­oxy-5,6-ep­oxy-3,5′-dihy­droxy-6′,7′-dide­hydro-5,6,7,8,5′,6′-hexa­hydro-β,β-caroten-8-one (Bonnett *et al.*, 1969 ▸). Later NMR studies of allenic carotenoids of the fuco­xanthin series and of geometrical isomers of fuco­xanthin provided a detailed solution-state foundation for the connectivity and *E*/*Z* geometry (Englert *et al.*, 1990 ▸; Hashimoto *et al.*, 2002 ▸; Haugan *et al.*, 1992 ▸). The first total synthesis of optically active fuco­xanthin and halocynthiaxanthin further established a stereochemical reference for the natural product (Yamano *et al.*, 1995 ▸).

Historically, however, single-crystal X-ray structure determination of fuco­xanthin has proved exceptionally challenging. In his review of physicochemical and synthetic studies on carotenoids, Moss (1979 ▸) reported that Sheldrick had partially solved the fuco­xanthin structure sufficiently to identify the two polyene chains, but that refinement was arrested at *R* ≃ 25%, even with the use of restraints. This early attempt is a striking indication of the difficulty posed by a long, weakly absorbing, highly conjugated and conformationally flexible carotenoid containing epoxide, hy­droxy, acet­oxy and allenic functionalities. Motivated by this unresolved crystallographic problem, and mindful of Sheldrick’s lasting legacy in crystallographic structure solution and refinement (Sheldrick, 2008 ▸; Sheldrick, 2015*b* ▸; Usón & Herbst-Irmer, 2025 ▸), we reinvestigated fuco­xanthin using modern single-crystal growth, crystal-handling and diffraction techniques.

Crystallographic precedents for other carotenoids demonstrate both the promise and the difficulty of this class of mol­ecules. The crystal structures of unbound astaxanthin, canthaxanthin and zeaxanthin allowed detailed discussion of end-group conformation, conjugated-chain geometry and the effect of crystalline environment on carotenoid colour (Bartalucci *et al.*, 2007 ▸). More recent work on lutein crystal forms has illustrated the importance of polymorphism and solvation in carotenoid packing and stability (Guo *et al.*, 2021 ▸). These studies show that ordered lattices can be obtained for extended polyene pigments, but also that solvatomorphism, conformational flexibility and end-group disorder are central features of carotenoid crystal chemistry (Fig. S1 in the supporting information).

Fucoxanthin has also been observed in pigment–protein com­plexes, including the fuco­xanthin chloro­phyll a/c-binding protein from *Phaeodactylum tricornutum*. The crystal structure deposited as PDB entry 6a2w contains seven fuco­xanthin ligands bound within the protein scaffold (Wang *et al.*, 2019 ▸). More recent cryo-EM structures of fuco­xanthin chloro­phyll a/c-binding assemblies have expanded the structural context of protein-bound fuco­xanthin mol­ecules (Kato *et al.*, 2024 ▸). These macromolecular models are indispensable for understanding binding-site geometry, but ligand geometries in protein structures are influenced by dictionary restraints, local density quality and limited ligand resolution. A high-resolution small-mol­ecule structure is therefore needed as an independent metrical and stereochemical reference.

The structure reported here represents the first small-mol­ecule single-crystal X-ray crystallographic determination of fuco­xanthin as an isolated mol­ecular com­pound, rather than as a restrained ligand model in a protein environment. The structure contains two crystallographically independent fuco­xanthin mol­ecules and two diethyl ether mol­ecules in the asymmetric unit. It provides direct crystallographic parameters for the allenic cyclo­hexyl end group, the conjugated polyene/ketone segment and the 5,6-ep­oxy ionone end group, and it allows com­parison of the solid-state conformations with a protein-bound A86 fuco­xanthin conformer from PDB entry 6a2w.

## Experimental

### Crystallization

Fucoxanthin (TOPPAN Holdings Inc., >98%) was dissolved in anhydrous diethyl ether at 300 K. Slow evaporation under nitro­gen at 300 K yielded aggregates of dark-orange needles. Crystals lose solvent within minutes in air; all manipulations were performed under ether vapour and crystals were mounted in Fomblin-Y and flash-cooled to 100 K.

### Data collection and refinement

Crystal data, data collection and structure refinement details are summarized in Table 1 ▸. Data were collected at the BL02B1 beamline of SPring-8 using synchrotron radiation (λ = 0.4132 Å) and a PILATUS 3X CdTe 1M detector (Dectris). The frame images were converted to SFRM format using *Henkankun-R* (Shikama *et al.*, 2018 ▸). Data integration, structure solution with *SHELXT* (Sheldrick, 2015*a* ▸) and subsequent refinement with *SHELXL* (Sheldrick, 2015*b* ▸) were carried out using the *APEX3* (Bruker, 2016 ▸) suite. H atoms were treated using a combination of independent and constrained refinement. The H atoms attached to the terminal allene C atoms (H13 and H55) were located in difference Fourier maps and refined freely. The four hy­droxy H atoms H93–H96 were treated so as to allow torsional refinement of the O—H directions while retaining the standard O—H distance; the remaining H atoms were placed in calculated positions and refined using riding models. The Flack parameter refined to −0.2 (10) from 8327 Parsons quotients; be­cause the structure contains only light atoms and was measured using high-energy radiation, the anomalous signal is not sufficient for an independent determination of the absolute structure. The absolute hand was therefore assigned by reference to the known natural product (3*S*,5*R*,6*S*,3′*S*,5′*R*,6′*R*) stereochemistry. The largest residual-density peak (0.55 e Å^−3^) is located adjacent to the C89—C90 end of the O14-containing diethyl ether mol­ecule and may reflect minor unresolved positional disorder of C90; no split disorder model was introduced.

## Results and discussion

### Crystal data, mol­ecular contents and atom labelling

Fucoxanthin crystallizes as a diethyl ether monosolvate, C_42_H_58_O_6_·C_4_H_10_O, in the space group *P*1 at 100 K. The asym­metric unit contains two crystallographically independent fuco­xanthin mol­ecules and two diethyl ether mol­ecules (Figs. 1 ▸ and 2 ▸). In the crystallographic atom labelling used here, the first fuco­xanthin mol­ecule, fucoA, com­prises C1–C42 and O1–O6; the second mol­ecule, fucoB, com­prises C43–C84 and O7–O12. The solvent mol­ecules are O13/C85–C88 and O14/C89–C92. This atom-labelling convention is used throughout the discussion. For the chemical discussion, the mol­ecule is divided into three domains: (i) the allenic cyclo­hexyl end group, C1–C13/O1–O3 in fucoA and C43–C55/O7–O9 in fucoB; (ii) the conjugated polyene/ketone segment, C12–C33/O4 in fucoA and C54–C75/O10 in fucoB; and (iii) the 5,6-ep­oxy ionone end group, C32–C42/O5/O6 in fucoA and C74–C84/O11/O12 in fucoB. These structural domain names are intentionally descriptive rather than formal IUPAC substituent names; they are suitable for structural com­parison of fuco­xanthin and related xanthophylls.

### Solvate-assisted crystal packing

Although the diethyl ether mol­ecules are not part of the chromophore, they are not merely incidental void-filling solvent. Refinement identified four O—H⋯O hy­dro­gen-bonding contacts (Table 2 ▸). In fucoA, O1—H93 and O6—H94 donate to ether atoms O14 and O13, respectively. In fucoB, O7—H95 donates to O14, whereas O12—H96 donates to acetate carbonyl atom O9 of a translation-related fucoB mol­ecule, generating a fucoB⋯fucoB chain. Thus, atom O14 acts as an acceptor for two O—H⋯O contacts (Table 2 ▸ and Fig. 3 ▸). These contacts are best described as weak-to-moderate O—H⋯O hy­dro­gen-bonding inter­actions (Desiraju & Steiner, 1999 ▸). The ether mol­ecules therefore appear to assist the organization of the otherwise highly hydro­phobic carotenoid framework rather than forming strong hy­dro­gen-bonded motifs. Similar packing behaviour has been noted in related carotenoid crystal structures, where weak O—H⋯O and C—H⋯O contacts, polyene-chain stacking, end-group disorder and solvent inclusion collectively influence the packing arrangements (Bartalucci *et al.*, 2007 ▸; Guo *et al.*, 2021 ▸).

### Allenic cyclo­hexyl end group

The allenic cyclo­hexyl end group is well defined in both crystallographically independent mol­ecules. In fucoA, the allene is C1=C12=C13, whereas the corresponding unit in fucoB is C43=C54=C55. The metrical parameters listed in Table 3 ▸ show two short C=C distances and an almost linear C=C=C angle in both mol­ecules, as expected for an allenic unit with mutually orthogonal π-systems. The acet­oxy substituent is attached through atom O2 in fucoA and through atom O8 in fucoB. Although the local acet­oxy geometry is closely com­parable in the two mol­ecules, the orientation of this substituent differs between fucoA and fucoB, indicating that this end group is one of the conformationally responsive regions in the crystal. Thus, the allenic cyclo­hexyl end group is not simply a rigid appendage, but one of the main conformationally responsive regions in the crystal (Fig. S3 in the supporting information).

### Conjugated polyene/ketone segment

The central conjugated polyene/ketone segment displays the bond-length alternation characteristic of a carotenoid π-system. The selected distances in Table 3 ▸ show that the formal C=C bonds are consistently shorter than the inter­vening C—C bonds in both fucoA and fucoB, while the ketone group is repre­sent­ed by a short C32—O4 bond in fucoA and the corresponding C74—O10 bond in fucoB. The representative torsion angles are close to 180° over most of the conjugated chain, confirming that the chromophoric framework is largely all-*E* and nearly planar in the solid state. This direct crystallographic observation is consistent with the solution-state formulation derived from NMR studies of fuco­xanthin and its geometrical isomers (Englert *et al.*, 1990 ▸; Hashimoto *et al.*, 2002 ▸; Haugan *et al.*, 1992 ▸). The small deviations from coplanarity are concentrated near the terminal-ring junctions rather than in the central polyene itself. Accordingly, the principal conformational variability in the crystal is associated with the terminal domains rather than with the central conjugated chain.

### 5,6-Ep­oxy ionone end group

The 5,6-ep­oxy ionone end group is also well resolved in both independent mol­ecules. In fucoA, the epoxide is C34—O5—C35, whereas the corresponding epoxide in fucoB is C76—O11—C77. The C—O distances and three-membered-ring angles listed in Table 3 ▸ are closely similar for the two mol­ecules, indicating that the intrinsic epoxide geometry is conserved. In contrast, the torsion angles that connect this end group to the polyene/ketone segment differ between fucoA and fucoB. The local stereochemical arrangement of the epoxide-bearing ring is therefore retained, but its orientation relative to the chromophoric chain is variable. This behaviour is consistent with the role of ionone-type terminal rings as conformationally flexible end groups in xanthophyll and carotenoid crystal structures (Bartalucci *et al.*, 2007 ▸; Guo *et al.*, 2021 ▸).

### Stereochemical implications and absolute structure

The present structure defines the relative stereochemistry of fuco­xanthin directly in the solid state. In the atom labels used here, the stereochemically significant tetra­hedral centres are C2, C4, C34, C35 and C37 in fucoA, and C44, C46, C76, C77 and C79 in fucoB, together with the allenic stereogenic axis C1=C12=C13 in fucoA and C43=C54=C55 in fucoB. The two crystallographically independent mol­ecules have the same connectivity and relative stereochemical arrangement. The relative configuration is illustrated in Fig. S1 in the supporting information; the R*/S* notation there denotes relative configuration only.

Because the structure contains only light atoms and was measured at short wavelength, the refined Flack parameter [−0.2 (10)] is not decisive for absolute structure determination. The absolute hand of the model was therefore assigned by reference to the established natural product and synthetic stereochemical formulation rather than from anomalous scattering determination alone. The refined Flack parameter is statistically consistent with zero and hence with the assigned hand, although its large standard uncertainty precludes an independent absolute structure determination (Thompson & Watkin, 2011 ▸). The crystallographic model provides a direct coordinate-based validation of the relative stereochemical arrangement inferred from degradative chemistry, solution-state NMR and total synthesis (Bonnett *et al.*, 1969 ▸; Englert *et al.*, 1990 ▸; Haugan *et al.*, 1992 ▸; Yamano *et al.*, 1995 ▸).

### Comparison with protein-bound A86 fuco­xanthin in PDB entry 6a2w

The protein-bound fuco­xanthin-A86 ligand in PDB entry 6a2w provides a useful com­parison with a biologically bound fuco­xanthin conformer, but its ligand geometry should not be used as a small-mol­ecule metrical standard. The deposited structure was determined at 1.80 Å resolution and contains seven fuco­xanthin mol­ecules in the protein scaffold (Wang *et al.*, 2019 ▸). Such ligand models reflect the combined effects of local electron density, refinement restraints and protein binding inter­actions. Consequently, the appropriate com­parison is conformational rather than metrical.

A graph-based atom correspondence followed by Kabsch superposition of fuco­xanthin-A86 residue 302 against fucoA gives a heavy-atom root-mean-square deviation of 0.619 Å (Schrödinger, 2026 ▸). The corresponding com­parison with fucoB is provided in Fig. S2. The com­parison is intended to show conformational similarity and terminal-domain flexibility rather than to com­pare individual bond lengths or angles (Fig. 4 ▸). The representative A86/fucoA overlay shows that the largest deviations are localized around the terminal-ring junctions and the acet­oxy/allene-containing end-group region, whereas the conjugated polyene framework is largely con­served. This com­parison supports a central conclusion of the small-mol­ecule structure: the polyene chain provides a relatively conserved structural spine, while the terminal domains accommodate substantial conformational adjustment.

## Conclusion

The single-crystal structure of fuco­xanthin diethyl ether monosolvate represents the first small-mol­ecule single-crystal X-ray structure of this allenic ep­oxy xanthophyll. The structure confirms the constitution and relative stereochemical arrangement previously inferred from degradative chemistry, solution-state NMR and total synthesis, while providing direct metrical parameters for the allene, epoxide, acet­oxy substituent, conjugated ketone and polyene chain. The presence of two crystallographically independent mol­ecules shows that the polyene core is conformationally conserved, whereas both terminal end groups retain appreciable flexibility in the solid state. The diethyl ether mol­ecules participate in the packing through O—H⋯O hy­dro­gen-bonding contacts. Comparison with protein-bound A86 fuco­xanthin in PDB entry 6a2w further indicates that the small-mol­ecule structure supplies a more reliable metrical reference, while the protein-bound conformer shows the same principal pattern of conformational variation, namely, conservation of the polyene spine and flexibility of the terminal domains. The structure therefore resolves a long-standing crystallographic problem in carotenoid chemistry and supplies a benchmark for future structural discussion of fuco­xanthin and related allenic ep­oxy xanthophylls.

## Supplementary Material

Crystal structure: contains datablock(s) I, global. DOI: 10.1107/S2053229626008946/oj3044sup1.cif

Structure factors: contains datablock(s) I. DOI: 10.1107/S2053229626008946/oj3044Isup2.hkl

Figs S1, S2 and S3 for additional supporting figures. DOI: 10.1107/S2053229626008946/oj3044sup3.pdf

Supporting information file. DOI: 10.1107/S2053229626008946/oj3044Isup4.cml

CCDC reference: 2584036

Additional supporting information:  crystallographic information; 3D view; checkCIF report

## Figures and Tables

**Figure 1:**
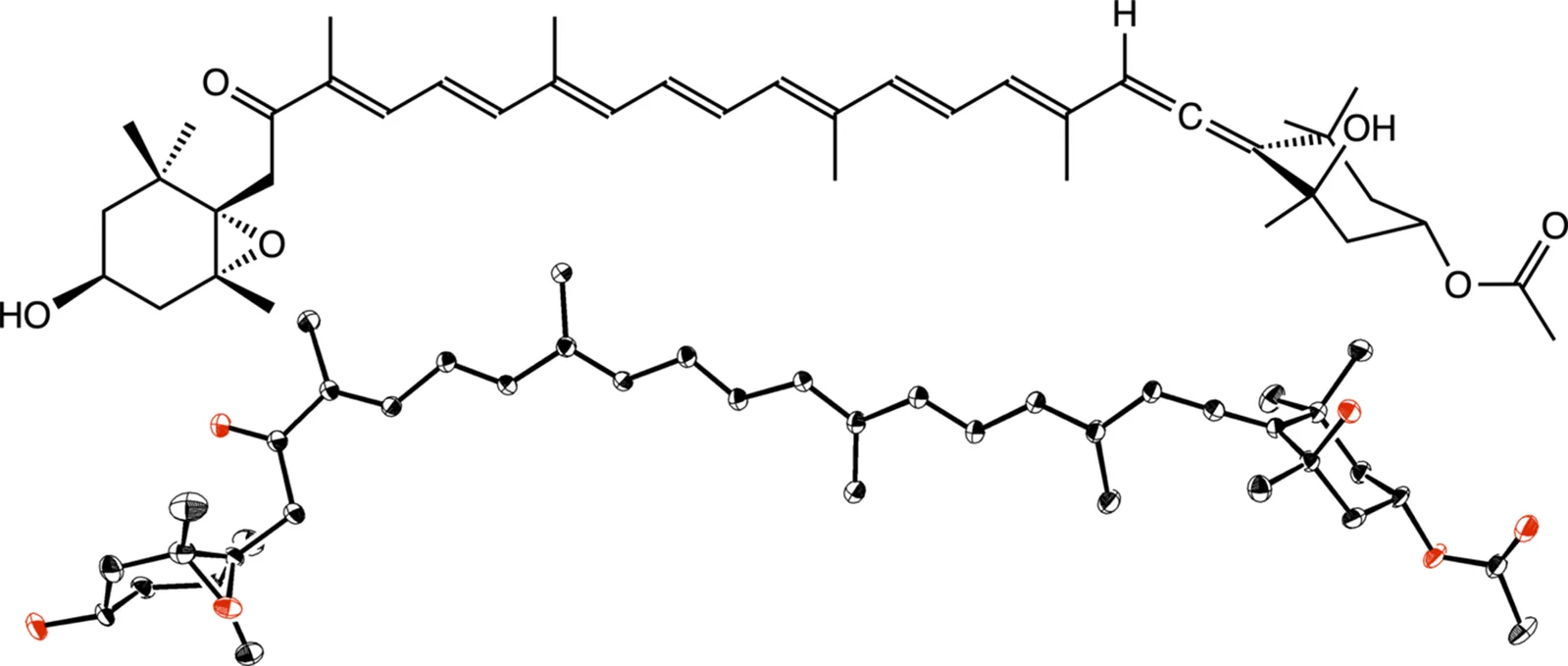
Chemical structure of fuco­xanthin (top) and the crystal structure of fucoA (bottom) in the fuco­xanthin diethyl ether monosolvate, with displacement ellipsoids drawn at the 50% probability level.

**Figure 2:**
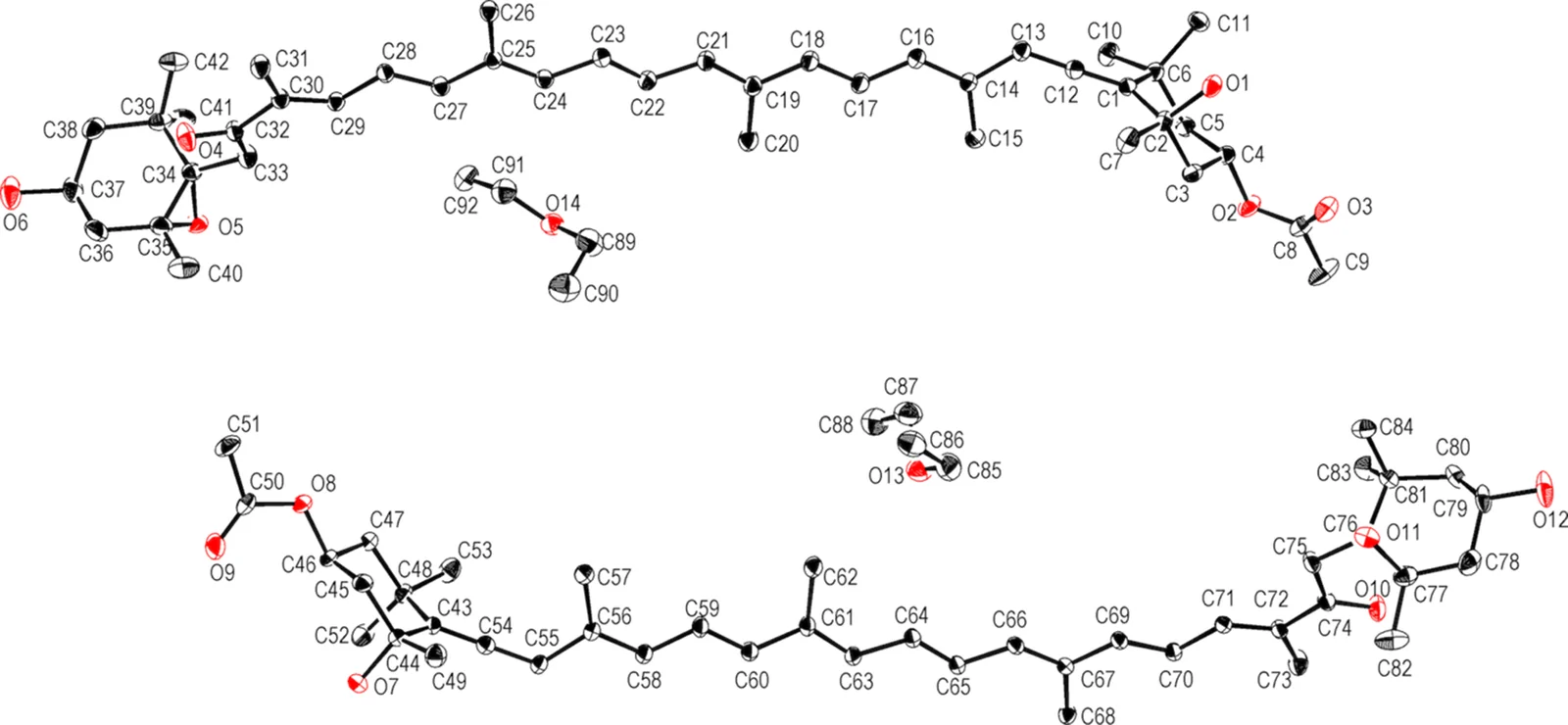
Packing diagram of fuco­xanthin diethyl ether monosolvate with the atom labels.

**Figure 3:**
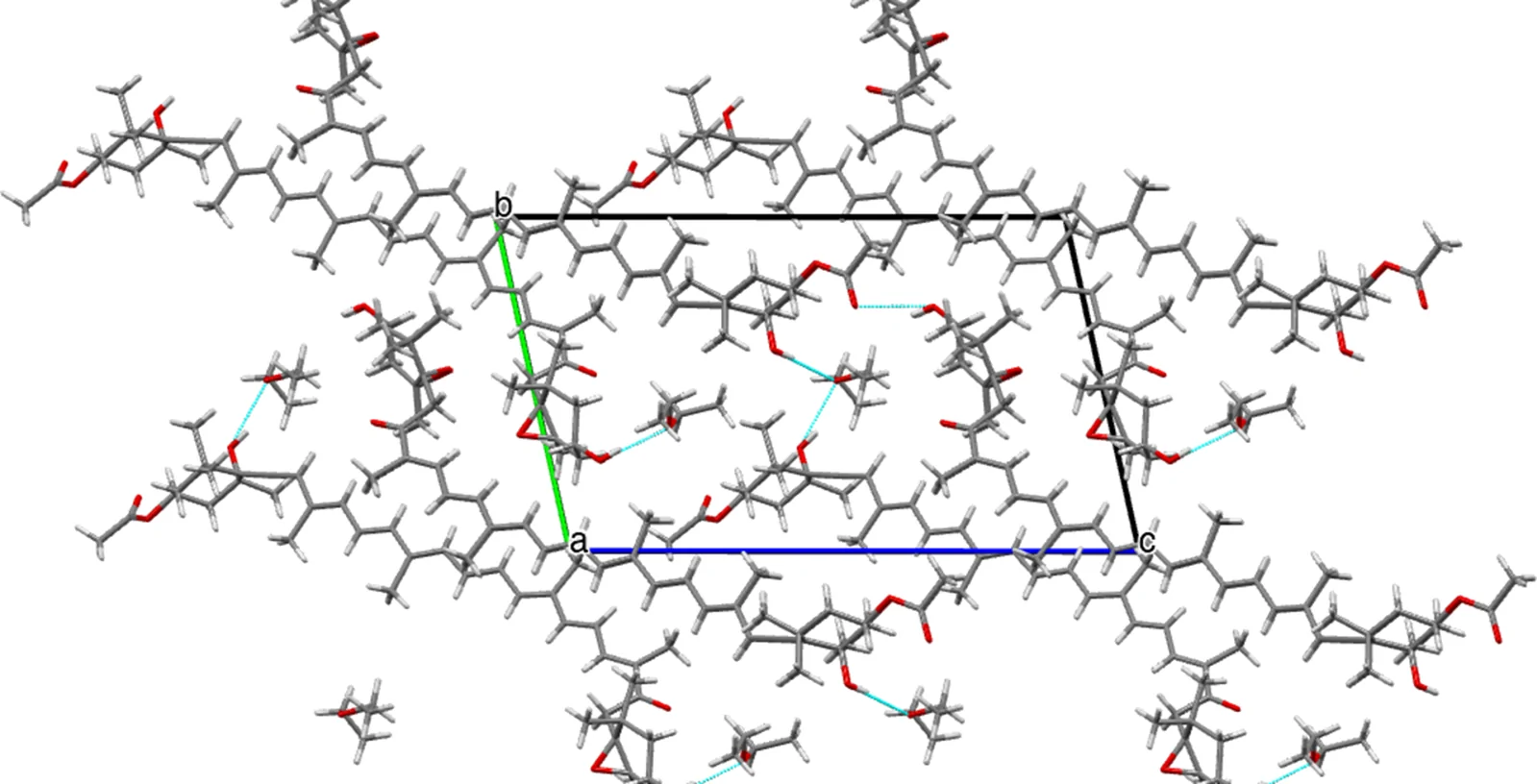
O—H⋯O hy­dro­gen-bond contacts between fuco­xanthin hy­droxy groups and diethyl ether monosolvate.

**Figure 4:**
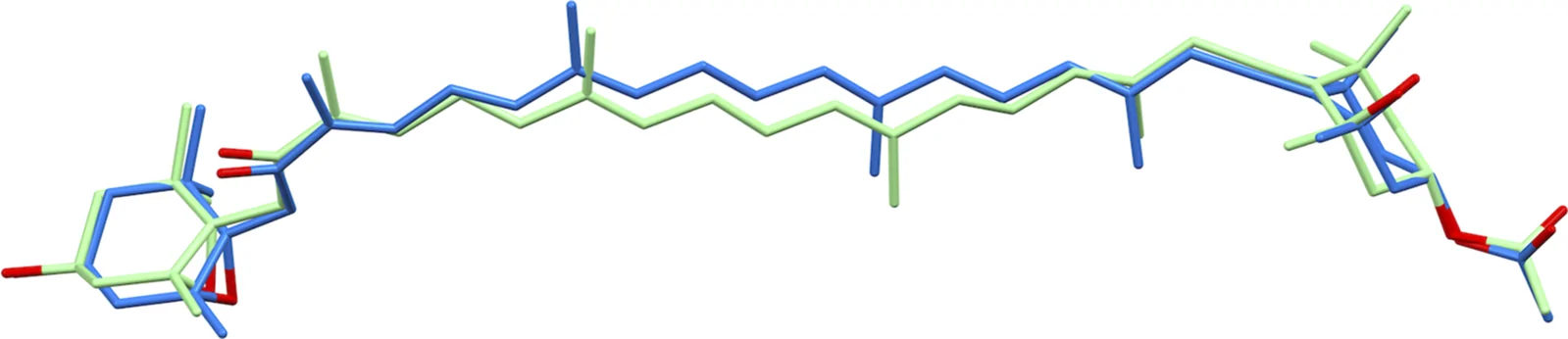
Overlay of protein-bound fuco­xanthin-A86 residue 302 from PDB entry 6a2w with fucoA from the present small-mol­ecule structure. A86 is shown in green, fucoA in blue and O atoms in red.

**Table 1:** Experimental details

Crystal data	
Chemical formula	C_42_H_58_O_6_·C_4_H_10_O
*M*_r_	733.00
Crystal system, space group	Triclinic, *P*1
Temperature (K)	100
*a*, *b*, *c* (Å)	7.9162 (9), 13.1285 (16), 21.944 (3)
α, β, γ (°)	101.178 (2), 99.094 (2), 96.991 (2)
*V* (Å^3^)	2181.7 (4)
*Z*	2
Radiation type	Synchrotron, λ = 0.4132 Å
μ (mm^−1^)	0.03
Crystal size (mm)	0.01 × 0.01 × 0.01

Data collection	
Diffractometer	BL02B1 beamline of SPring-8 using a PILATUS 3X CdTe 1M detector (Dectris)
Absorption correction	Multi-scan (*SADABS*; Bruker, 2016 ▸)
*T*_min_, *T*_max_	0.790, 1.000
No. of measured, independent and observed [*I* > 2σ(*I*)] reflections	50529, 19428, 18495
*R*_int_	0.062
(sin θ/λ)_max_ (Å^−1^)	0.649

Refinement	
*R*[*F*^2^ > 2σ(*F*^2^)], *wR*(*F*^2^), *S*	0.049, 0.133, 1.04
No. of reflections	19428
No. of parameters	993
No. of restraints	3
H-atom treatment	H atoms treated by a mixture of independent and constrained refinement
Δρ_max_, Δρ_min_ (e Å^−3^)	0.55, −0.32
Absolute structure	Flack *x* determined using 8327 quotients [(*I*^+^) −(*I*^−^)]/[(*I*^+^) + (*I*^−^)] (Parsons *et al.*, 2013 ▸)
Absolute structure parameter	−0.2 (10)

Computer programs: *APEX3* (Bruker, 2016 ▸), *SAINT* (Bruker, 2016 ▸), *SHELXT2014* (Sheldrick, 2015*a* ▸), *SHELXL2014* (Sheldrick, 2015*b* ▸) and *SHELXTL* (Sheldrick, 2008 ▸).

**Table 2:** Hydrogen-bond geometry (Å, °) The C91—H91*A*⋯O10^ii^ contact is included for completeness; the discussion in the text focuses on the O—H⋯O network.

*D*—H⋯*A*	*D*—H	H⋯*A*	*D*⋯*A*	*D*—H⋯*A*
O1—H93⋯O14^i^	0.84	2.13	2.898 (2)	153
O6—H94⋯O13^ii^	0.84	2.07	2.907 (3)	174
O7—H95⋯O14^iii^	0.84	2.06	2.899 (2)	173
O12—H96⋯O9^iv^	0.84	2.20	2.945 (3)	147
C91—H91*A*⋯O10^ii^	0.99	2.60	3.589 (3)	173

Symmetry codes: (i) 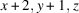; (ii) 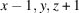; (iii) 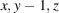; (iv) 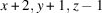.

**Table 3:** Selected geometrical parameters (Å, °) for the three structural domains of fuco­xanthin

Parameter/domain	fucoA	fucoB
**Allenic cyclo­hexyl end group**		
Allene C=C	C1—C12 1.308 (3); C12—C13 1.314 (3)	C43—C54 1.300 (3); C54—C55 1.317 (3)
Allene angle	C1—C12—C13 177.3 (2)	C43—C54—C55 178.4 (2)
Acet­oxy C—O /O—C/C=O	C4—O2 1.460 (3); O2—C8 1.352 (3);	C46—O8 1.468 (2); O8—C50 1.336 (3);
	C8—O3 1.199 (3)	C50—O9 1.208 (3)
		
**Conjugated polyene/ketone segment**		
Mean formal C=C in polyene chain	1.359; range 1.354–1.368	1.358; range 1.355–1.366
Mean inter­vening C—C in polyene chain	1.449; range 1.425–1.484	1.449; range 1.430–1.477
Ketone C=O	C32—O4 1.222 (3)	C74—O10 1.219 (3)
Representative polyene torsion angles	C12—C13—C14—C16 177.9 (2);	C54—C55—C56—C58 −171.5 (2);
	C13—C14—C16—C17 −179.3 (2);	C55—C56—C58—C59 176.8 (2);
	C16—C17—C18—C19 −177.0 (2);	C58—C59—C60—C61 177.5 (2);
	C21—C22—C23—C24 177.6 (2)	C63—C64—C65—C66 −175.7 (2)
		
**5,6-Ep­oxy ionone end group**		
Epoxide C—O	C34—O5 1.461 (3); C35—O5 1.457 (3)	C76—O11 1.463 (3); C77—O11 1.453 (3)
Epoxide angle	C34—O5—C35 60.90 (14)	C76—O11—C77 60.85 (14)
Hy­droxy C—O	C37—O6 1.430 (3)	C79—O12 1.428 (3)
Terminal-ring junction torsion angles	C30—C32—C33—C34 −163.4 (2);	C72—C74—C75—C76 −179.1 (2);
	C32—C33—C34—C35 −77.8 (3);	C74—C75—C76—C77 −71.3 (3);
	C33—C34—C35—C36 153.8 (2)	C75—C76—C77—C78 153.2 (2)

Note: formal C=C and inter­vening C—C averages were calculated from the crystallographically defined conjugated polyene segment, excluding the allene and the terminal saturated ring bonds.
