# Supplementary material for: Crystal structure of fuco­xanthin: resolving a long-standing crystallographic challenge
Source: Acta Crystallogr C Struct Chem. 2026 Sep 5;82(Pt 10):627–33. doi: 10.1107/S2053229626008946 (PMC13637801; doi:10.1107/S2053229626008946)
Supplement: Supplementary file 1 [file c-82-00627-sup3.pdf]

STRUCTURAL CHEMISTRY

Volume 82 (2026)

Supporting information for article:

Crystal structure of fucoxanthin: resolving a long-standing crystallographic challenge

Yui Wakasa and Mao Minoura

Electronic Supplementary Information for:

Crystal structure of fucoxanthin: resolving a long-standing crystallographic challenge

Yui Wakasa[a] and Mao Minoura[a*]

[a] Department of Chemistry, College of Science, Rikkyo University, 3-34-1 Nishi-Ikebukuro, Toshima-ku, Tokyo 171-8501, Japan.

minoura@rikkyo.ac.jp

Table of Contents

Figure S1. Chemical structure of fucoxanthin, canthaxanthin, lutein, astaxanthin, and zeaxanthin with CSD-code references. For fucoxanthin, the absolute hand shown is assigned from the established natural-product stereochemistry; the asterisk denotes relative configuration.

Figure S2. Overlay of fucoA (blue) with fucoB (green) from the present small-molecule structure.

Figure S3. Overlay of fucoA (blue) and fucoB (green), aligned using the 5,6-epoxy ionone end-group core, showing progressive divergence along the polyene chain and the greatest difference at the allene-containing end.

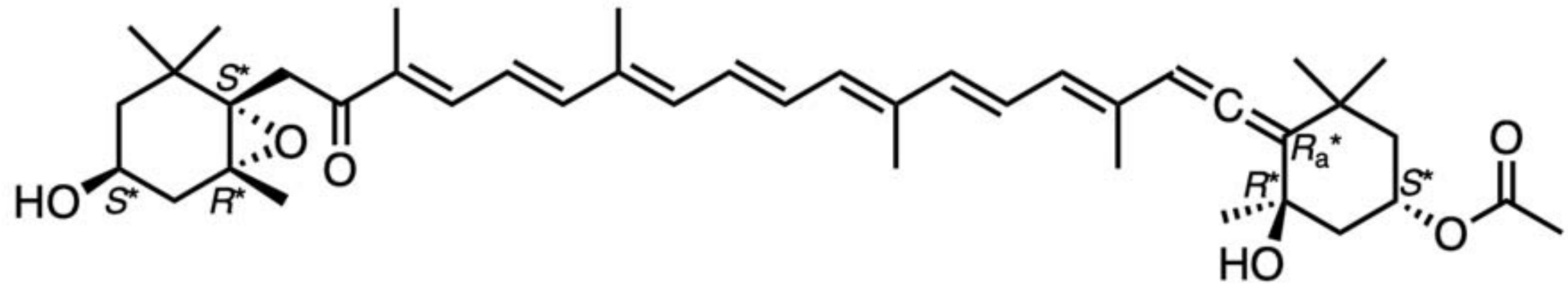

Fucoxanthin: FCOXTN (No crystal data)

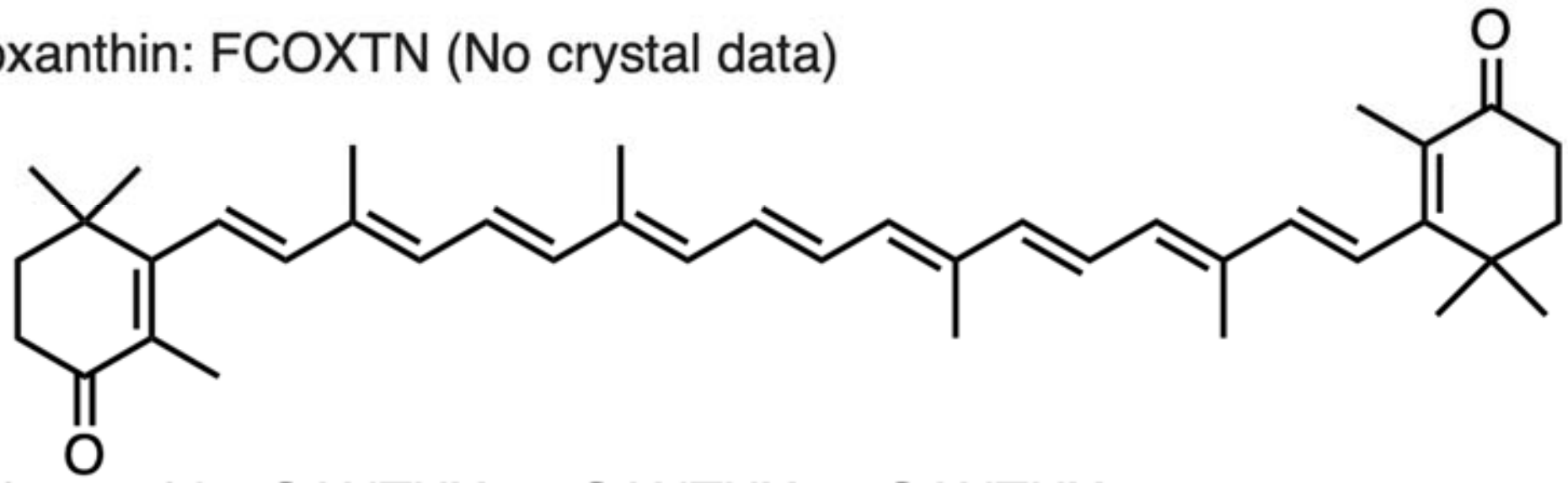

Canthaxanthin: CANTHX10, CANTHX11, CANTHX12

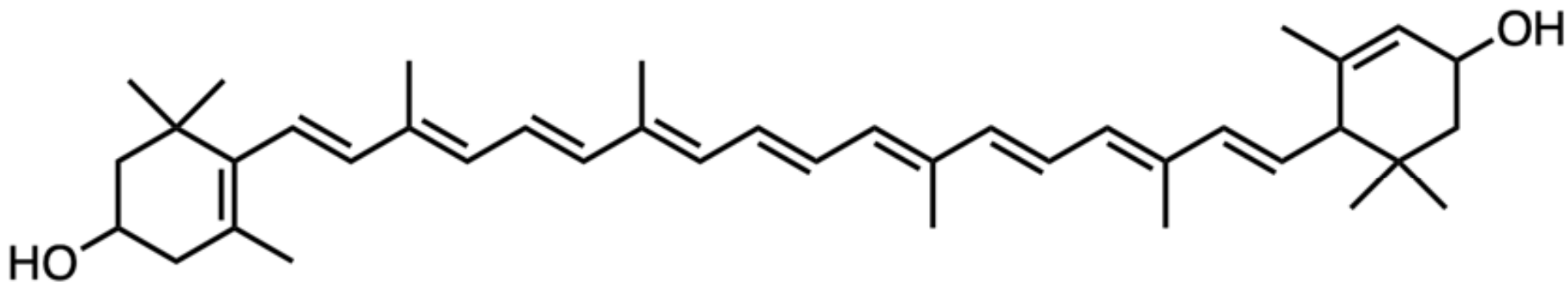

Lutein: AKEGEF, AKEQAL

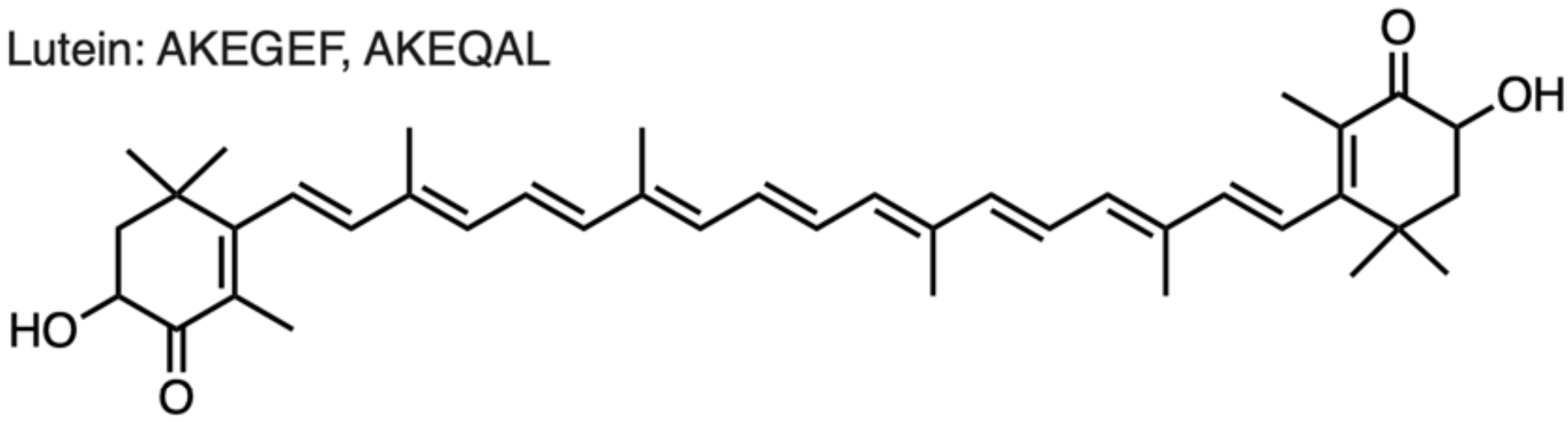

Astaxanthin: BAHYEP, BAHYEP01, XEZJAO, XEZJES

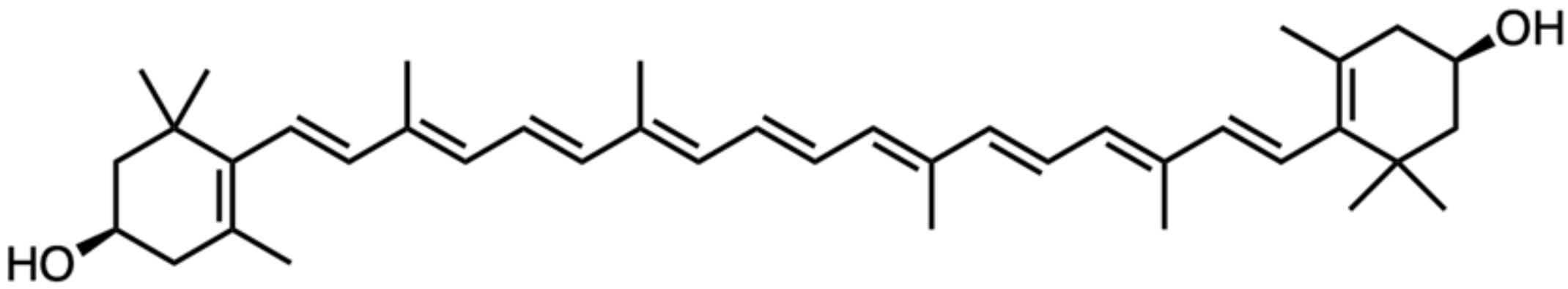

Zeaxanthin: XEZJIW

Figure S1. Chemical structure of fucoxanthin, canthaxanthin, lutein, astaxanthin, and zeaxanthin with CSD-code references. For fucoxanthin, the absolute hand shown is assigned from the established natural-product stereochemistry; the asterisk denotes relative configuration.

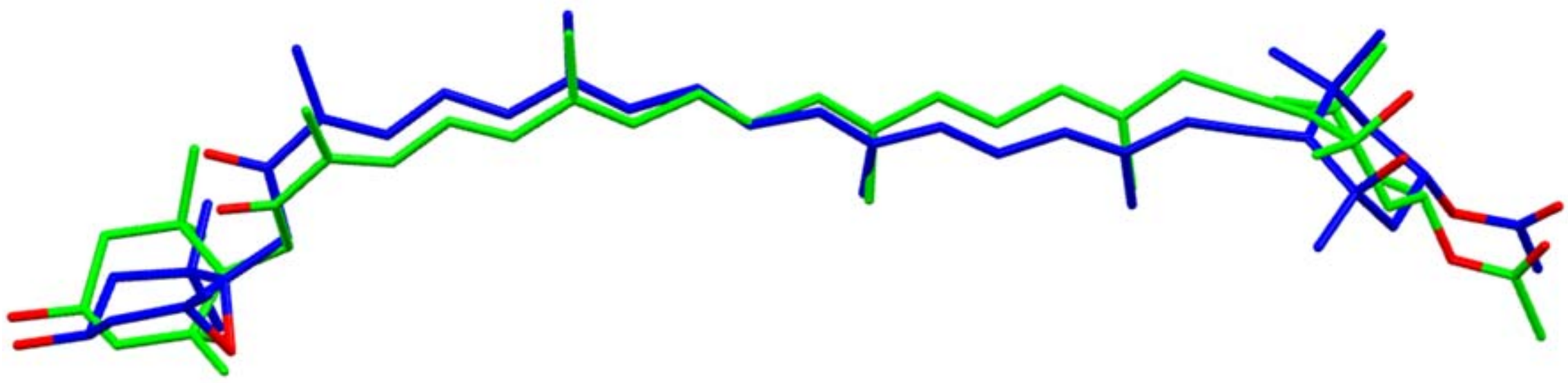

Figure S2. Overlay of fucoA (blue) with fucoB (green) from the present small-molecule structure.

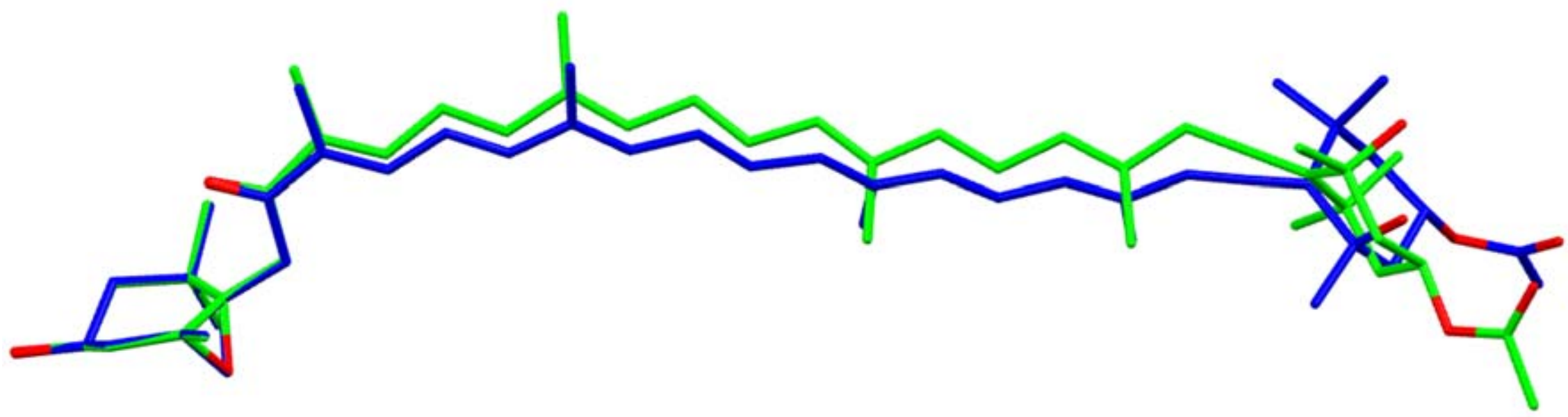

Figure S3. Overlay of fucoA (blue) and fucoB (green), aligned using the 5,6-epoxy ionone end-group core, showing progressive divergence along the polyene chain and the greatest difference at the allene-containing end.
